# Stenosis triggers spread of helical *Pseudomonas* biofilms in cylindrical flow systems

**DOI:** 10.1038/srep27170

**Published:** 2016-06-07

**Authors:** David R. Espeso, Ana Carpio, Esteban Martínez-García, Victor de Lorenzo

**Affiliations:** 1 Centro Nacional de Biotecnología, CSIC, Madrid, 28049, Spain; 2 Universidad Complutense, Matematica Aplicada, Madrid, 28040, Spain

## Abstract

Biofilms are multicellular bacterial structures that adhere to surfaces and often endow the bacterial population with tolerance to antibiotics and other environmental insults. Biofilms frequently colonize the tubing of medical devices through mechanisms that are poorly understood. Here we studied the helicoidal spread of *Pseudomonas putida* biofilms through cylindrical conduits of varied diameters in slow laminar flow regimes. Numerical simulations of such flows reveal vortical motion at stenoses and junctions, which enhances bacterial adhesion and fosters formation of filamentous structures. Formation of long, downstream-flowing bacterial threads that stem from narrowings and connections was detected experimentally, as predicted by our model. Accumulation of bacterial biomass makes the resulting filaments undergo a helical instability. These incipient helices then coarsened until constrained by the tubing walls, and spread along the whole tube length without obstructing the flow. A three-dimensional discrete filament model supports this coarsening mechanism and yields simulations of helix dynamics in accordance with our experimental observations. These findings describe an unanticipated mechanism for bacterial spreading in tubing networks which might be involved in some hospital-acquired infections and bacterial contamination of catheters.

Numerous bacterial species form biofilms, which are multi-cell assemblies attached to surfaces and held together by an extracellular polymeric matrix^1^,^2^,^3^. Living in a biofilm, bacteria become extremely tolerant to antibiotics and disinfectants^4^,^5^. Biofilms found on medical equipment such as catheters and other implants are the cause of numerous hospital-acquired infections, including bloodstream, urinary tract and prosthesis-associated infections^6^,^7^,^8^. In industrial environments, biofilms cause considerable damage in scenarios as diverse as food poisoning, biofouling and ventilation systems^9^,^10^,^11^,^12^, making their presence a substantial economic and technical problem. Nonetheless, biofilms also aid in many biotechnological applications and wastewater treatment^11^,^13^,^14^. Some of the most destructive effects of biofilm formation are the complications brought about by *Pseudomonas* species in immunocompromised patients following colonization of catheters and other medical devices^15^. This is not limited to *Pseudomonas aeruginosa*, a pathogenic bacterium that can be fatal when it affects critical organs^16^,^17^. Infections by the habitual soil-dweller and plant-root colonizer *P. putida* are also documented, linked to the use of incorrectly sterilized solutions and implant of catheters or drainage tubes^18^,^19^,^20^,^21^, including cases of sepsis and bacteremia^22^,^23^,^24^,^25^. Most *P. putida* strains (e.g., the reference strain KT2440) lack the genes that prompt virulence^26^, which makes their handling safer. In our hands, this provided us with an optimal model to study some mechanical and physical aspects of biofilm development^27^,^28^. By combining numerical models with wet experimentation, we have inspected the spread of *P. putida* biofilms on the inner surfaces of circular tubes, the usual shape of many medical devices that penetrate the patient’s body^29^,^30^. To this end, we established a simple hydraulic tubing arrangement that combined diverse diameters and types of junctions in which drip or pulsatile flow moved the fluid. *P. putida* cells were then injected at distinct sites of such tubes and the biofilm formation process examined at joints and connectors. As shown below, bacterial filaments formed that evolved to wrap around the inner walls of the tubes in a precise helical pattern. Our experiments, together with supporting *in silico* simulations, demonstrate that the physical setting brought about by pulsatile flows facilitated colonization of the tubes in the system. These data not only document and explain mechanistically the formation of helical biofilms in cylindrical hydraulic systems, but also ask for a reconsideration of the type of hydraulic flows adopted for liquid delivery in medical devices.

## Results

### Helical biofilm pattern develops in different setups of tubes

**Figure 1:**
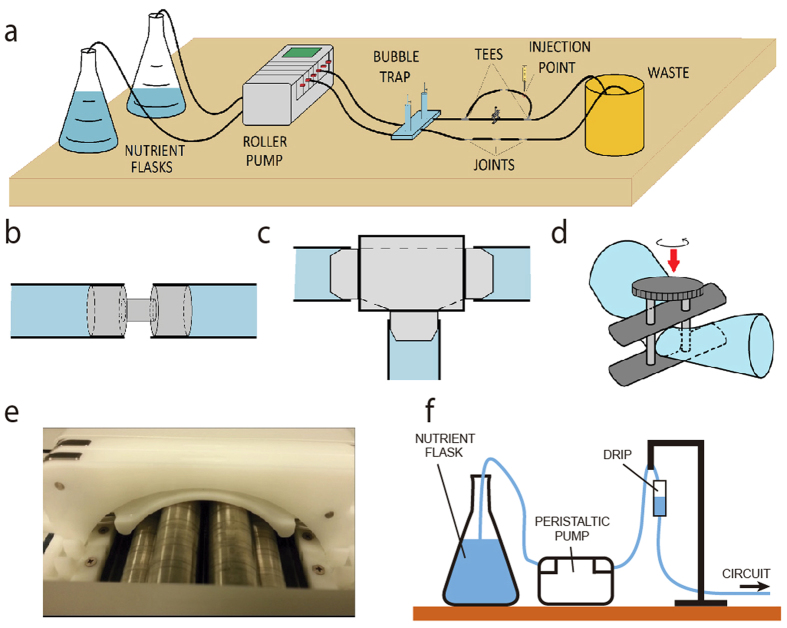
Experimental setup description. (**a**) Sketch of an experimental setup. Diameter variations in the tubes: (**b**) adaptor, (**c**) T-junction, (**d**) vertically oriented clamp. (**e**) Detail of the roller pump ISMATEC IP-C 8 used in the experiments. (**f**) Scheme of the device used for the drip flow experiment.

The millifluidic flow system adopted for inspection of biofilm development in cylindrical silicone tubings is sketched in Fig. 1a. The setup allowed inoculation of defined numbers of *P. putida* cells at distinct sites, as explained in the Materials and Methods section. A first series of experiments were run with the reference strain *P. putida* KT2440 and flow rates of 0.15*–*0.45 ml/min (average linear velocities ranging within 0.8–2.3 mm/s for 2 mm diameter tubes) at room temperature for a maximum of 10 days. The Reynolds number (a dimensionless number expressing the ratio of inertial to viscous forces defined as Re = ρvD/μ, where ρ, v, μ represent fluid density, velocity and viscosity, whereas D is the tube diameter) was thus in the range 1.5–4.7. Hydraulic circuit designs were customized to perturb the hydrodynamic flow conditions in the silicone tubes (Supplementary Fig. S1a–j). We used connectors to alter the diameter (internal diameter of 1–2 mm), T-junctions to split or merge conduits, and clamps to partially occlude tubes by deforming cylindrical geometry (Fig. 1b–d). Biofilm filaments appear systematically past stenoses and angles caused by adaptors, junctions and clamps.

**Figure 2:**
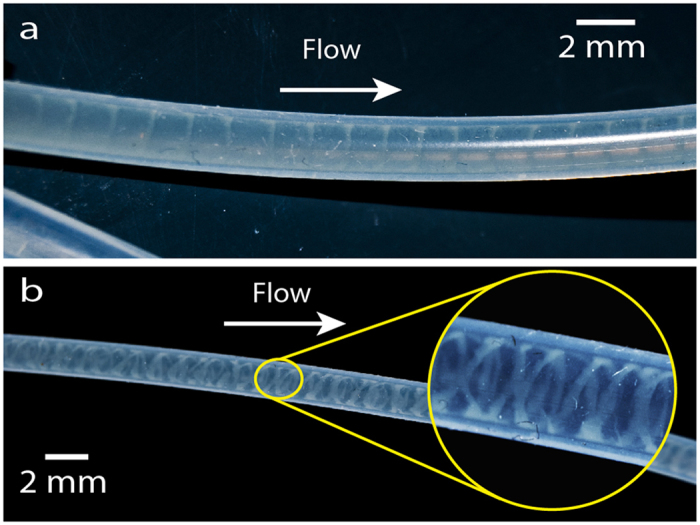
Wild-type *P. putida* KT2440 helical biofilms wrapped inside a 2 mm inner diameter silicone tube. Differences in local geometry or curvature prompt the formation of (**a**) Single helix or (**b**) overlapping helices. Photographs were taken after 4 days of continuous pulsatile pumping generated by a roller multiport pump at a flow rate of 0.45 ml/min. Helices are visible to the naked eye. Brightness and contrast were adjusted to enhance the images.

**Figure 3:**
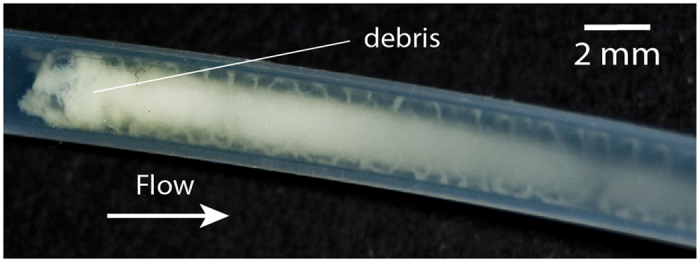
An advancing air bubble destroyed a helical biofilm, and debris flowing downstream showed that helices are located near the tube wall. Fully developed helices reside near the tube walls. Brightness and contrast were adjusted to enhance the image.

We noted the formation of long biofilm threads wound on the walls in different parts of the tubing network (Fig. 2a,b). The helix adapts its pitch to the tube diameter, with larger pitches for larger diameters (Supplementary Fig. S2). Helices tended to fill the entire tube length in which they grew, with an overall length of several centimeters or decimeters (Supplementary Fig. S3). Fully developed helices resided near the tube walls and did not interfere with primary flow, as Fig. 3 shows. Specific local geometry (clamps, junctions, adaptors) and curvature can alter the chances of bacterial attachment to the surface, which will generate one or several biofilm seeds; single (Fig. 2a) or multiple (Fig. 2b) helical threads are observed as a consequence.

**Figure 4:**
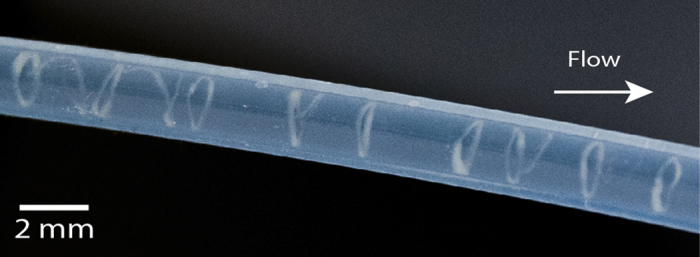
Image showing a broken helical thread split into sequences of rings. Brightness and contrast were adjusted to enhance the image.

These coiled structures are brittle and small perturbations, such as vibrations or air bubbles, could destroy the helix or split the thread into a series of rings (Fig. 4). When the flow stopped, the structure collapsed slowly and dissolved; if flow was restored, the helical patterns reappeared. Developing filaments only attached to the wall at certain points; accelerated Supplementary Movie S1 shows slow downstream motion of the helical thread past a clamp. Still images show that these threads formed as a result of a dynamic process that shaped the increasing biomass in time and space; structures twisted with time but were also displaced downstream, generating a train of perturbations along the tube.

### Helical patterns arise from an elastic instability process

The development of the helical biofilms observed in our experiments is a spontaneous mechanical process that reshapes an expanding biofilm thread with excess length constrained into a tube of cylindrical section and fixed length. The helical pattern arises as a natural mechanical equilibrium state that minimizes the elastic energy of the thread^31^,^32^. Constraints forbidding straight thread equilibrium such as an excess length or a source of twist favor the onset and coarsening of helical instabilities. A straight thread of a certain length confined in a shorter tube can evolve into a helicoidal structure if an appropriate perturbation is applied^31^,^33^,^34^.

**Figure 5:**
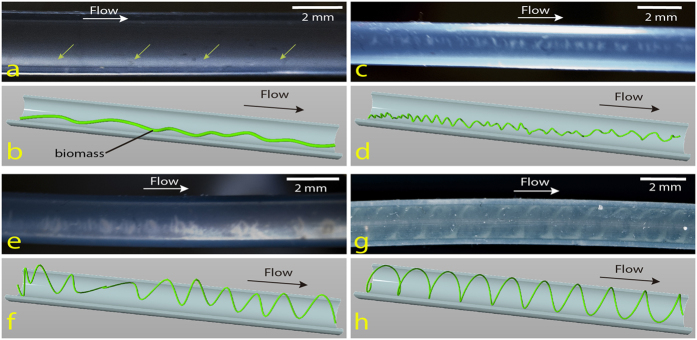
Scheme showing stages of helix formation over time (illustrations supported by experimental images selected from different experiments because of their clarity). (**a–b**) Nucleated straight biofilm thread (green arrows) flows downstream. (**c–d**) Helical instability bends the biofilm thread to shape an initial wavy pattern. (**e–f**) Biomass accumulation and subsequent deformation foster radial expansion of the wavy pattern resulting in a sequence of large bent loops. (**g–h**) Tube radius constrains helix enlargement and molds the thread into the final helical shape. Brightness and contrast were adjusted to enhance the images.

**Figure 6:**
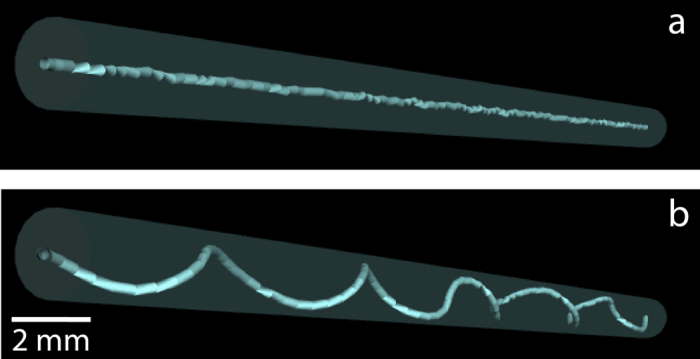
Numerical solution and description of the three-dimensional discrete filament model. Helix evolution showing: (**a**) Emergence of the helical instability and (**b**) coarsening due to the increase in length.

In order to predict some characteristics of helical pattern formation we used a discrete filament model^35^ to study the dynamics of threads in these geometries (see Methods and Supplementary Text). Figure 5 illustrates helix formation and evolution, and Figs 6 and S4 together with Supplementary movies S2 and S3 show computational description and results of our model in qualitative agreement with experiments. Randomly perturbed threads (Fig. 5a,b) with increased length develop into tiny helices (see Figs 5c,d and 6a). The increase in length is first distributed along the entire structure by bending of the thread out of the plane, to form a helix-like train of perturbations (helical loops). These perturbations compete with each other to lengthen the thread, which leads to formation of larger loops at the expense of others that disappear (Fig. 6b, analogous to Fig. 5e,f). The thread finally wraps around the interior walls of the tube, if its length permits. The final helical shape is determined by thread length and tube radius (Fig. 5g,h; Supplementary Fig. S4). Once it is wrapped around the tube, the pitch depends on the tube radius r_tube_ and the excess length L_helix_ − L_tube_, where L_tube_ is the length of the tube and L_helix_ the length of the filament wrapped around it. There is a length restriction to generate an effective helix, imposed by the size of the tube. A thread of length L_helix_ will wrap around the tube forming *k* steps of pitch L_tube_/*k* provided that: L_helix_^2^ ~ L_tube_^2^ + 4π^2^r_tube_^2^ *k*^2^. Our simulations in Fig. 6 and Supplementary Fig. S4 provide final helical pitches of 5 mm for a 2 mm tube, in the range of observed pitches. The intermediate stages of helix evolution are influenced by the elastic parameters of the thread, the enlargement rate of the thread as well as by its length and the twist at any of the thread edges, which can increase slowly with time, see Supplementary Text.

The mechanical properties of the biomass define the evolution of the helical pattern as our model and experiments (see next below) suggest: biofilms are heterogeneous materials composed of interface-associated colonies of bacteria embedded in an extracellular matrix; the bacteria harden the matrix and modify its response^36^. Moreover experimental observations characterized biofilms as viscoelastic materials^37^,^38^,^39^ whose mechanical properties change dynamically in response to cellular processes and interaction with the environment^40^,^41^.

**Figure 7:**
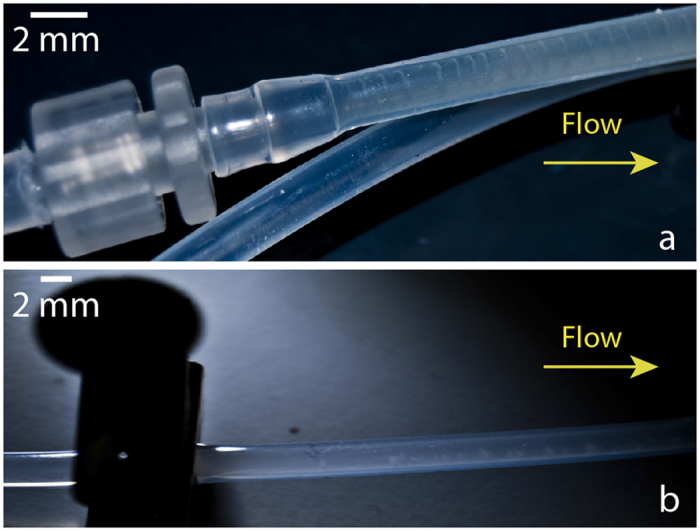
Effect of the stenoses in helix structure and evolution. Replacing (**a**) circular connectors by (**b**) vertically oriented clamps (see Fig. 1d) deforms the symmetry in the tube cross-section, forcing the nucleated thread to expand along the bottom of the tubes and to adopt an asymmetric and irregular shape. Brightness and contrast were adjusted to enhance the images.

The helical pattern was observed to progress along the nucleated filaments in different ways in different experiments: the formation of helical patterns was not only downstream a constriction (with its initial nucleation point at stenoses as explained above, see Fig. 7a,b, Supplementary Fig. S5) but also upstream an obstruction (when a filament previously nucleated at an injection point finally hits a surface, see Supplementary Fig. S6a,b). In the last case, the biomass thread flowing downstream undergoes a deceleration when it is injected into the waste flask and drops onto the surface, causing a coiling effect^42^,^43^,^44^,^45^ that amplifies the helical instability at the thread. Thus our experiments indicate that both biomass growth and thread coiling at its extremes are two sources of perturbations which foster the helical pattern formation, in accordance with our theoretical results.

### Stenoses foster nucleation of biofilm threads and modify their evolution

**Figure 8:**
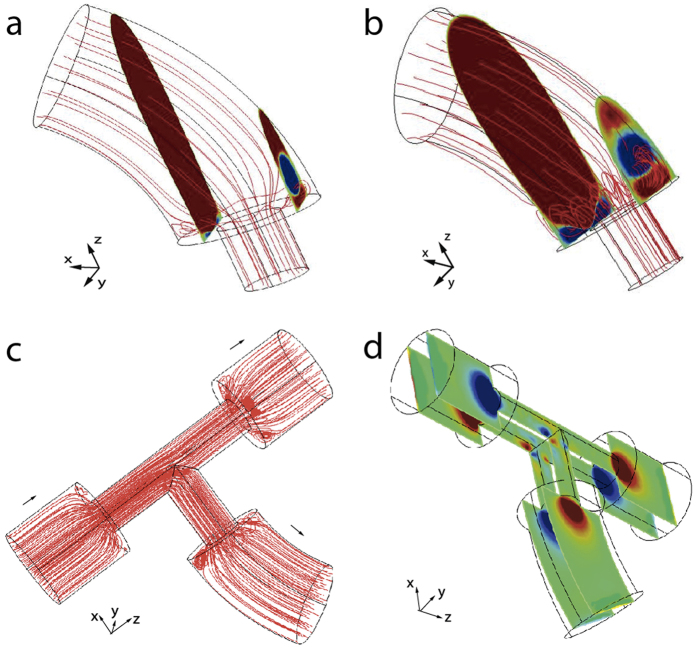
Numerical plot of fluid streamlines at different stenosis points. (**a**) Steady drip flow and (**b**) pulsatile flow at a fixed time through a tube adaptor. The size of the vortical region in (**b)** expands and shrinks periodically over time. Colored slices indicate the direction of the *x* component of velocity. (**c**) Streamline and (**d**) *y* component of fluid velocity chart inside a T-Junction. Note in addition to the vortices after the stenoses, the secondary vortices formed at the corners by the streamlines in (**c**).

The helical biofilm patterns in Figs 2, 3, 4 and Supplementary Fig. S2 appeared at T-junction exits (Fig. 1c) and in splitting or merging conduits. Similar patterns were observed in straight lines joined by diameter adaptors or clamps (Fig. 1b,d), but also in straight unconstricted tubes (Supplementary Fig. S6). What causes filament formation? The initial bacterial inoculum results in an initial attachment of bacteria and subsequent biofilm formation near the inoculation points. Bacteria reproduce within biofilms and produce extracellular polymeric substances (EPS), a polymer-based mixture formed with extracellular DNA, proteins and polysaccharides. Part of this substance admixed with cells (biomass) is eroded by fluid shear force and borne downstream following the streamlines. The process finally prompts the formation of a long thread as observed in Fig. 5a. This mechanism is reminiscent of that for biofilm thread nucleation at corners^38^,^47^. Moreover, since the Reynolds number was small (about 1–4, at least 1000 times smaller than the onset of turbulent regime) the flow was laminar and we noticed that stenoses were favoured points in the tubing network for an enhanced nucleation process. To test our hypothesis we performed a numerical simulation, where we solved incompressible Navier-Stokes equations to visualize the flow streamlines in tubes and stenoses, using the same parameters and geometries applied in the experiments. Standard finite element solvers showed the formation of small localized vortices at narrowings (Fig. 8a,b) and junctions (Fig. 8c,d). Biomass is eventually trapped in the vortices^48^,^49^,^50^ and driven to the walls, where it adheres and generates additional biofilm seeds^38^. This process sustained in time shapes biofilm seeds into one or several filaments, that flow downstream through the central part of the tubes (Fig. 5a,b). The resultant number of nucleated helical filaments will finally depend of the specific combination of parameters affecting the local hydrodynamics at stenosis points (tube cross-section size and shape, curvature, dynamics of the flow).

The geometry of the stenoses also exerts an influence on the subsequent helix formation dynamics. This relationship between helix thread shape and stenosis geometry effect was confirmed by replacing circular connectors (Fig. 7a) with clamps (Fig. 7b) to deform the tube cross-section asymmetrically. An incipient helical filament was formed (Fig. 7b) comparable to the helix of small radius and pitch in Fig. 5c. Clamps broke the symmetry, and biofilm filaments tended to spread along the bottom of the horizontal tubes (Supplementary Fig. S5a), which delayed helix formation for 3 days (from 7 to 10 days with the strain *P. putida* KT2440). Besides providing mechanisms for biofilm nucleation, narrowings may affect biofilm evolution in additional ways. An already formed straight biofilm thread that reaches a stenosis is strained and altered by velocity changes. This generates a train of perturbations that propagates downstream with the filament and is amplified by elastic mechanisms evolving to a helical shape constrained by the walls.

### Fluid dynamics and biomass accumulation shift the timing of helix formation

Biofilm thread evolution in fluids has been described as an interplay between a fluid and an elastic structure^46^,^51^, an hypothesis that best explains our observations. Therefore we investigated what was the role of the fluid and the biomass nature in helical pattern formation.

To set fluid in motion through the conduits, we used a roller pump (Fig. 1e). Roller pumps are based on a peristaltic mechanism that strains the fluid-containing duct with rollers to induce a pulsatile forward flow^52^. Changes in roller rotation frequency yield different flow rates. The pump connection tube was periodically locally compressed by a roller train of 1.8 cm diameter to generate small fluid packs forced to advance in the direction of the rotor. We first tested the effect of the flow rate in helix formation. When we increased the flow rate in 2 mm-inner diameter tubes from 0.15 ml/min (mean fluid velocity 0.8 mm/s) to 0.45 ml/min (mean fluid velocity 2.38 mm/s) we observed a reduction in the formation time of the helicoidal pattern by 2–3 days using KT2440 strain (from 7 to 4–5 days).

Additionally we studied the effect of pump-generated flow dynamics in the helical pattern, as we were using a peristaltic pump with a defined pulsatile flow of two superimposed periods of ∼0.1 and 0.8 s, that might be linked to helicoidal particle trajectories in certain conditions^52^,^53^. We performed flow visualization tests using ink injection to follow streamlines as they pass through narrowings and T-junctions. In these we observed small vortices beyond the junctions followed by a typical laminar Poiseuille profile (see Supplementary movies S4 and S5 and Fig. S7), but no helicoidal trajectories. Moreover, the Dean number (De, a dimensionless number that is the product of the Reynolds number and the square root of the tube diameter to curvature ratio) is too low for the development of sustained spiral flows (for our typical assemblies, De < 0.1, about 1000 times smaller than the onset of helical flows)^54^,^55^. Finally we devised one experiment by adapting the layout to replace pulsatile flow with steady drip flow (Fig. 1f; Supplementary Fig. S1c,d). Helices appeared even in absence of this pulsatile flow, as expected for filament shapes developing elastic instabilities once a flow mechanism to create biofilm seeds is available. Pulsatile flow seems to facilitate the formation of biofilm seeds due to enhanced recirculation past the stenoses (Fig. 8b). To determine the relevance of biomass accumulation, we used a second strain, *P. putida* mt-2 harboring the catabolic pWW0 plasmid that also confers the ability to produce more biofilm^26^,^56^,^57^. In our experimental setting (Supplementary Fig. S1a,b; initial OD_600_ = 0.2, flow rate 0.15 ml/min), *P. putida* mt-2 helices were observed after 7 days incubation, while 10 days were needed for KT2440, which suggests that biomass availability is relevant in helix evolution.

In further experiments we designed hydraulic circuits to specifically increase or decrease the amount of available biomass formed by *P. putida* mt-2 in the tubing network. In the former case (Supplementary Fig. S1g,h) we connected a 500 ml Erlenmeyer flask with a saturated culture. In the later case (Supplementary Fig. S1e,f) we changed the material of the tubes to diminish the attachment of bacteria onto the tubes by replacing silicone tubing by PVC tubes, which has delayed the attachment of some bacterial strains because of its enhanced hydrophobicity^58^. In the former case helices appeared in 24 h (instead of 3–4 days), whereas in the latter the helical pattern was completely suppressed. These results indicate a strong relationship between biomass amount accumulated in the tubes and the temporal window in which the helical pattern is formed.

## Discussion

The observation of helical biofilms in pulsatile and drip flows defines new possibilities to study biofilm spreading mechanisms. Helical biofilms appear to be the outcome of flow processes that occasionally drive cells and extracellular material to the tube walls, combined with mechanical deformation that twists biomass filaments confined within the tubes. Since diameter variations in conduits are prevalent in medical equipment, industrial systems and natural environments, one can expect that these helical structures are widespread. We experimentally observed the emergence of helical instabilities on long threads following the streamlines, and monitored the coarsening of helical filaments from the onset of instability to late stages in which they wrap around the tube interior. Helices arise as a purely physical outcome that distributes excess length in the thread of bacterially generated biomass, as illustrated by discrete filament models. This study increases our knowledge of the ability of bacteria to propagate in tube networks and suggests further works focused on describing the potential consequences that this phenomenon might cause when it develops in medical and industrial equipment.

## Methods

### Bacterial strains and growth conditions

All strains used in this study were fluorescently labeled derivatives of *P. putida*, the parental strain *P. putida* mt-2^56^ and the TOL plasmid-free *P. putida* KT2440 59. The fluorescent derivative strains were obtained by site-specific insertion at a chromosomic intergenic region of miniTn*7*-derivatives with a constitutive promoter driving the expression of GFP^60^. Bacteria were grown overnight in M9 minimal medium^61^ supplemented with 0.2% (w/v) glucose as carbon source plus 5 *μg/ml* gentamicin. Optical density of cultures was adjusted to OD_600_ of 0.2 and 250 *μl* of the suspension was injected into the network with a 29G × 1/2″ − 0.33 × 12 mm syringe (Terumo). The inoculation point was approximately set in the middle tube section (Fig. 1a). Bacteria were allowed to attach to the tubes (45 min) and flow was set in motion at 0.15 ml/min. When the hydraulic circuit had one pumping line, final flow rate was 0.15 ml/min; in the case of three lines, final flow rate was 0.45 ml/min (see Supplemenatary Fig. S1).

The entire network was sterilized by pumping a 0.2% (w/v) sodium hypochlorite solution at 1.6 ml/min during 1 h, washed with sterilized H_2_O, and buffered with M9 medium as above. Alternatively, polycarbonate flow cells connected to the network (BioCentrum-DTU) were used as initial bacterial inoculation and attachment sites. Flow cells were covered with a 24 × 50 mm cover slip (Menzel-Glaser) and sealed with silicone glue.

### Flow assay setup and imaging

We prepared several network architectural configurations to study the influence of variables on helix pattern formation (Supplementary Fig. S1a–j). The network contained a 2 L Erlenmeyer flask filled with fresh M9 medium, a multiport roller pump that sucked fluid from the nutrient reservoir (ISMATEC IP-C 8), a bubble trap, and a waste tank. The circuit elements were joined with peroxide crosslinked silicone tubes (VWR) with an internal diameter of either 1 and/or 2 mm. Tubes were connected using polypropylene T-junctions and adaptors (EW-30623–66, 1/16″, Cole Parmer) with a 0.9 mm inner diameter. Clamps (VWR screw compressor clamps) were used when needed. The pump was connected to the circuit with 1.65 inner diameter mm Tygon tubes (R3607, VWR). Images were acquired using a Nikon D60 camera with a AF-S Nikkor 18–55 mm lens. Some experimental pictures (indicated in their captions) were treated with Adobe Photoshop (Adobe Systems) to improve brightness and contrast to accentuate helices within the tubes. Time-lapse videos were filmed with a ScopeEye USB Microscope (NKS Tech Apps).

### Discrete filament model

**Figure 9:**
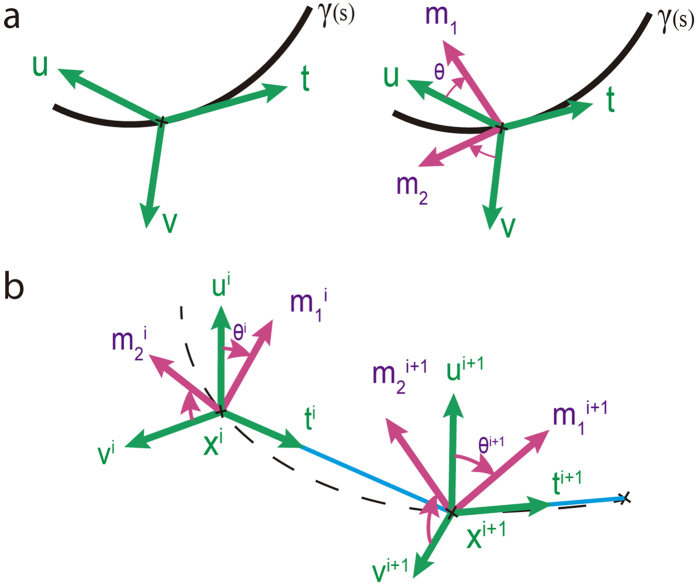
Centerline and material frame representing filament deformation. (**a**) Continuous description and (**b**) discrete description. The Bishop frame {**t**, **u**, **v**} defines the “rest orientation” at any point of the centerline. The material frame characterizing the local orientation {**t**(s), **m**_1_(s), **m**_2_(s)} is obtained rotating {**u**(s), **v**(s)} an angle *θ* around **t**(*s*). The centerline is discretized into a set of points {**x**_0_, **x**_1_, …, **x**_n+1_} and segments **e**^*i*^ = **x**_*i*+1_ *−* **x**_*i*_. A local orthonormal material frame {**t**^*i*^, **m**_1_^*i*^, **m**_2_^*i*^} is assigned to each point setting **t**^*i*^ = **e**^*i*^**/||e**^*i*^||, the unit tangent vector per edge.

Our model describes the dynamics of a growing biofilm thread exposed to the action of elastic bending and torsion forces. In filaments, one dimension (length) is much larger than the other (cross-section), which leads to description of filament evolution by a curve (the centerline) with an orientation frame {**t**, **m**_**1**_, **m**_**2**_} (the material frame, Fig. 9a), that captures the rotation of the material cross-sections at each point along the curve^62^,^63^. Stretching and bending are represented by deformation of the centerline, whereas twist is captured by orientation of the material frame. When deformations are small compared to the characteristic thread length, the stresses that affect cross-sections can be averaged and described as a force and moment acting on the centerline. While it neglects cross-section deformations, this approach describes the large-scale physical and geometrical properties of long, thin filaments. The filament is represented by a sequence of segments **e**^*i*^ = **x**_*i*+1_ *−* **x**_*i*_ joining points **x**_*i*_ and material frames {**t**^*i*^, **m**_1_^*i*^, **m**_2_^*i*^} (Fig. 9b). **t**^*i*^is the unit tangent vector per segment. The material frame vectors are obtained by rotating an angle *θ*^*i*^relative to the Bishop frame {**u**^*i*^, **v**^*i*^}, a reference frame with no twist (see Fig. 9 and Supplementary Text). Filament evolution is defined by variation of the angles **θ**^i^ and node positions **x**_*i*_.

Assuming inextensibility^46^, the total energy of the discrete filament is the sum of the bending and twist energies^35^:


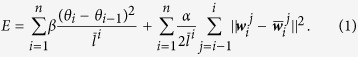


*α* and *β* are the bending and torsion modulus, respectively. The factor 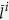 is related to the length of the segments in the undeformed configuration. The vectors 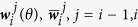 are material curvatures in the deformed and undeformed configurations, respectively. The material frame is updated in a quasistatic way^35^. By imposing 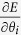 = 0 for all segments *i* not fixed by a boundary condition, a system of equations for the angles follows. The positions of the points are updated by solving the equations of motion for the centerline^35^:


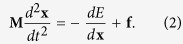


The inextensibility constraint is enforced by a projection manifold method^64^. f represents the external forces acting on the centerline^65^,^66^,^67^. Modeling the different mechanisms producing a length increase through additional streching terms in the energy is a complex task that may lead to unnecessarily stiff equations. Instead, we alternate the simulation of the bending and torsion processes with resetting steps in which the length between nodes or the number of nodes is varied at a fixed rate. More details in Supplementary Text I.

### Software

COMSOL Multiphysics was used for the flow simulations. The discrete filament model is implemented with MATLAB scientific software. The three dimensional evolution of the thread is visualized with virtual reality language VRML 2.0.

## Additional Information

**How to cite this article**: Espeso, D. R. *et al.* Stenosis triggers spread of helical Pseudomonas biofilms in cylindrical flow systems. *Sci. Rep.* **6**, 27170; doi: 10.1038/srep27170 (2016).

## Supplementary Material

Supplementary Information

Supplementary Video S1

Supplementary Video S2

Supplementary Video S3

Supplementary Video S4

Supplementary Video S5
