# Supplementary material for: Stenosis triggers spread of helical Pseudomonas biofilms in cylindrical flow systems
Source: Sci Rep. 2016 Jun 7;6:27170. doi: 10.1038/srep27170 (PMC4895183; doi:10.1038/srep27170)
Supplement: Supplementary Information [file srep27170-s1.pdf]

SUPPLEMENTARY INFORMATION

Title:
Stenosis triggers spread of helical *Pseudomonas* biofilms in cylindrical flow systems

Authors:

David R. Espeso, Ana Carpio, Esteban Martínez-García, and Víctor de Lorenzo*

*email : vdlorenzo@cnb.csic.es

SI text

The mathematical description of an elastic filament as a unidimensional centerline was discussed in References.[62,63] The rod is represented by an adapted framed curve $\Gamma = \{\gamma; \mathbf{t}, \mathbf{m}_1, \mathbf{m}_2\}$ (Fig. 9a). $\gamma(s)$ is the centerline parametrized by the arc length in $\mathbb{R}^3$. $\{\mathbf{t}(s), \mathbf{m}_1(s), \mathbf{m}_2(s)\}$ is the orthonormal material frame characterizing the local orientation. $\mathbf{t}(s) = \gamma'(s)$ is tangent to the curve. $\mathbf{t}' = \kappa$ is the curvature (normal) vector. The Bishop frame $\mathbf{B}(s) = \{\mathbf{t}, \mathbf{u}, \mathbf{v}\}$ defines the "rest orientation" at any point of the centerline. It is an adapted frame with zero twist, i.e., $\mathbf{u'} \cdot \mathbf{v} = -\mathbf{v'} \cdot \mathbf{u}$. Assigning the Bishop frame at $s = 0$ uniquely determines the remaining values by parallel transport. The twist of the thread at any point is implemented rotating $\Gamma(s)$ an angle θ with respect to $\mathbf{B}(s)$ around the plane defined by $\{\mathbf{u}(s), \mathbf{v}(s)\}$ (Fig. 9a):

$$\mathbf{m_1}(\theta) = \cos(\theta)\mathbf{u} + \sin(\theta)\mathbf{v}, \quad \mathbf{m_2}(\theta) = -\sin(\theta)\mathbf{u} + \cos(\theta)\mathbf{v}.$$

Following Ref.,[35] the centerline is discretized into a set of points $Q_n = \{\mathbf{x}_0, \mathbf{x}_1, ..., \mathbf{x}_{n+1}\}$ and segments joining them $\mathbf{e}^i = \{\mathbf{e}^0, \mathbf{e}^2, ..., \mathbf{e}^n\}$, where $\mathbf{e}^i = \mathbf{x}_{i+1} - \mathbf{x}_i$. A local orthonormal material frame $M^i = \{\mathbf{t}^i, \mathbf{m}_1{}^i, \mathbf{m}_2{}^i\}$ is assigned to each point (Fig. 9b). $\mathbf{t}^i = \frac{\boldsymbol{e}^i}{\|\boldsymbol{e}^i\|}$ is the unit tangent vector per edge. To construct a Bishop frame we set $\mathbf{u}^0 \perp \mathbf{t}^0$ and define $\mathbf{v}^0 = \mathbf{t}^0 \times \mathbf{u}^0$. The frames at other edges are found by parallel transport $\mathbf{u}^i = P_i(\mathbf{u}^{i-1})$, $\mathbf{v}^i = \mathbf{t}^i \times \mathbf{u}^i$. P_i are rotation matrices about the curvature binormal satisfying $P_i(\mathbf{t}^{i-1}) = \mathbf{t}^i$, $P_i(\mathbf{t}^{i-1} \times \mathbf{t}^i) = \mathbf{t}^{i-1} \times \mathbf{t}^i$. If $\mathbf{t}^{i-1} = \mathbf{t}^i$, P_i is the identity. The condition $\mathbf{u}^0 \perp \mathbf{t}^0$ must hold during the simulation. It is reestablished by parallel transport in time (instead of space). Let θ^i be the angles defining the material frames by rotation of the Bishop frames. The material frame vectors at each edge are:

$$\mathbf{m_1^i} = \cos(\theta^i)\,\mathbf{u}^i + \sin(\theta^i)\mathbf{v}^i, \; \mathbf{m_2^i} = -\sin(\theta^i)\,\mathbf{u}^i + \cos(\theta^i)\mathbf{v}^i$$

When the undeformed filament is straight and its elastic behaviour is isotropic, the elastic energy due to twisting and bending is given by the expression:

$$E = \sum_{i=1}^{n} \beta \frac{(\theta_i - \theta_{i-1})^2}{\bar{l}^i} + \sum_{i=1}^{n} \frac{\alpha}{2\bar{l}^i} \sum_{j=i-1}^{i} \left\| \boldsymbol{w}_i^j - \bar{\boldsymbol{w}}_i^j \right\|^2. \tag{1}$$

where α and β are the bending and torsion modulus, respectively. $\bar{l}^i$ is the length of the segments $\bar{\mathbf{e}}^i = \bar{\mathrm{x}}_{i+1} - \bar{\mathrm{x}}_i$ in a reference undeformed configuration $\{\bar{\mathbf{x}}_0, \bar{\mathbf{x}}_1, \dots, \bar{\mathbf{x}}_{n+1}\}$. The vectors $\mathbf{w}_i^j, \bar{\mathbf{w}}_i^j, j = i-1, i$, are material curvatures in the deformed and undeformed configurations, respectively:

$$\boldsymbol{w}_i^j = \left((\kappa\mathbf{b})_i \cdot \mathbf{m}_2^j, -(\kappa\mathbf{b})_i \cdot \mathbf{m}_1^j\right)^t, (\kappa\mathbf{b})_i = \frac{2\mathbf{e}^{i-1} \times \mathbf{e}^i}{\|\bar{\mathbf{e}}^{i-1}\|\|\bar{\mathbf{e}}^i\| + \mathbf{e}^{i-1} \cdot \mathbf{e}^i}$$

where $\kappa\mathbf{b}$ is the curvature binormal. The material frame is updated in a quasistatic way, imposing

$$\frac{\partial E}{\partial \theta_i} = 0,$$

for all segments i not fixed by a boundary condition.[35] The angle configuration minimizing the energy of the rod is determined by this system of equations. When the edges are clamped, we assign the material frame for $i = 0$ or $i = n$. Twist at the edges is fixed assigning the values of θ. For stress free ends no boundary condition is assigned.

The dynamics of the nodes is governed by Newton's second law

$$\boldsymbol{M}\frac{d^2\boldsymbol{x}}{dt^2} = -\frac{dE}{dx} + \boldsymbol{f}. \tag{2}$$

where f represents the external forces and $-\frac{dE}{d\mathbf{x}}$ the elastic forces, that can be evaluated once the angles are known.[35] $\mathbf{M}$ is the mass matrix, that is set equal to a multiple of the identity $\mathbf{M}$=m$\mathbf{I}$ for an isotropic thread. This system is integrated using a Verlet solver combined with a manifold projection method to enforce the inextensibility constraint for each segment, see Ref.[64] The force exerted by the fluid on the filaments can be evaluated following Ref.[65] Where a node hits the tube, a virtual force is applied to impose the spatial constraint generated by the presence of the wall (penalty method), keeping the node inside. Addition of biomass may be represented by increase of segment length. These equations can be coupled to the motion of bodies

placed at the edges of the thread following.[35]

Cells coupled to the filament are expected to be trapped rotating in the vortices formed at narrowings.[48–50] This is a source of twist, that can be introduced in the simulation following different procedures of increasing complexity: fixing a time dependent angle at the edge, coupling to spinning particles as in references.[66,67] The fluid force is modeled using Reference.[65]

For numerical purposes we nondimesionalize the equations (2). Choosing λ=1mm and T=1s as reference lengths and times, the change of variable $x = \lambda x'$, $t = T t'$ yields:

$$\frac{d^2\boldsymbol{x'}}{dt'^2} = -\frac{T^2}{m\lambda^2}\frac{dE}{d\boldsymbol{x'}} + \frac{T^2}{m\lambda}\boldsymbol{f} \tag{3}$$

Revising the definition of the energy E (1), this change brings about the controlling parameters $\alpha' = \frac{\alpha T^2}{m\lambda^3}$ and $\beta' = \frac{\beta T^2}{m\lambda^3}$. We are working with one dimensional filaments, therefore neglecting the cross-sections. A real cylindrical thread of density ρ, small radius r, and length $L >> r$, would be approximated in this framework by a discrete filament with N nodes and N-1 edges, with mass $m = \rho \pi r^2 L/(N-1)$. Knowing ranges of values of α', β' that lead to different types of numerical helical structures we might guess ranges for α, β provided that the density ρ and radius r of the thread are known.

We have performed series of tests in tubes with diameters 0.2, 0.5, 1, 2 mm and lengths 10, 40, 80 mm, and explored parameter ranges for which different helical dynamics are observed. Fig. 6 in the main text and Supplementary figure S4 and movie S2 and S3 are generated setting $\alpha' = 1.345$, $\beta' = 0.789$, $N = 100$. The tube diameter and length are 2 mm and 80 mm, respectively. Still images of Fig. 6 and S4 show snapshots of the simulations for 3200, 6300, 9600 and 16200 computational iterations. Simulations start from an originally straight thread in which the positions of the nodes have been randomly perturbed, resulting in a small length increase compared to the tube length (an excess around 0.49% of the tube length). The left edge of the thread is subject to smoothly increasing twist, reaching $7 \times \pi$ at the end. We alternate steps in which we solve (3) with steps in which we increase the length of the edges of the thread (edge length increase rate = $2 \cdot 10^{-5}$ mm/iteration) and reset the reference edges. The helix pitch reduces, as shown by Supplementary Fig. S4 and Supplementary movie S3. The final helix pitch is constrained by the excess length to one helical loop per 5 mm. Adding together the times during which equation (3) is numerically solved, and the time the biofilm thread needs to increase its length (due to biomass production, or other factors) by the specified amount, we would find the total evolution time. However, we lack experimental values for this later parameter.

When we couple the dynamics of the filament to the flow, the whole structure moves downstream very slowly as the helix develops, in a similar way to Supplementary movie S1. The twist is not really necessary for the thread to evolve into a helix. In tubes of smaller diameter, threads naturally evolve to helices wrapping around the walls provided the initial excess length allows it. However, the presence of twist improves the stability of the evolution as the length of the thread increases, avoiding knots and messy structures.

Supplementary figures

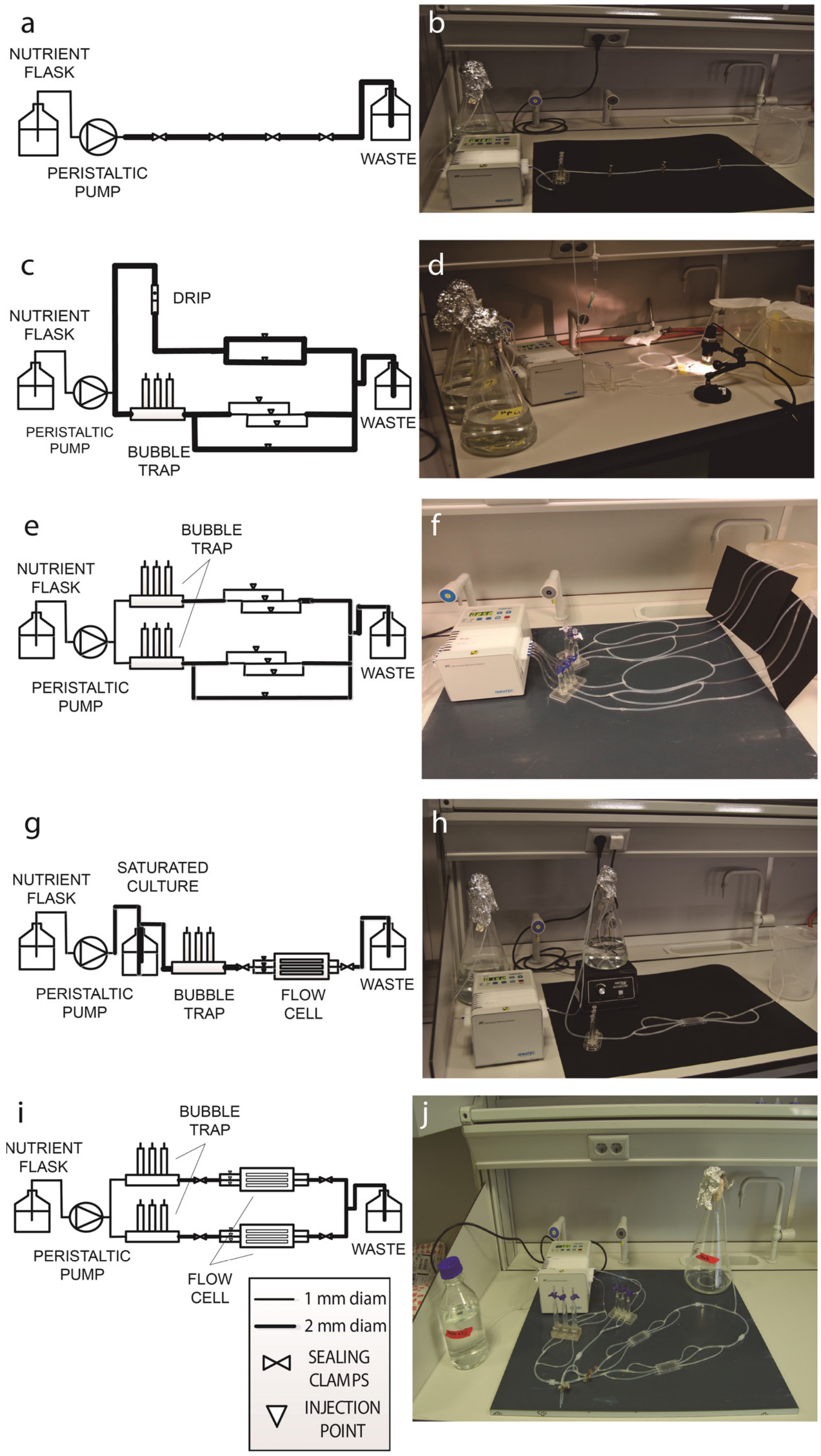

Figure S1. Several experimental setups were designed (**a**,**c**,**e**,**g**,**i**) and tested (**b**,**d**,**f**,**h**,**j**) to study helix formation.

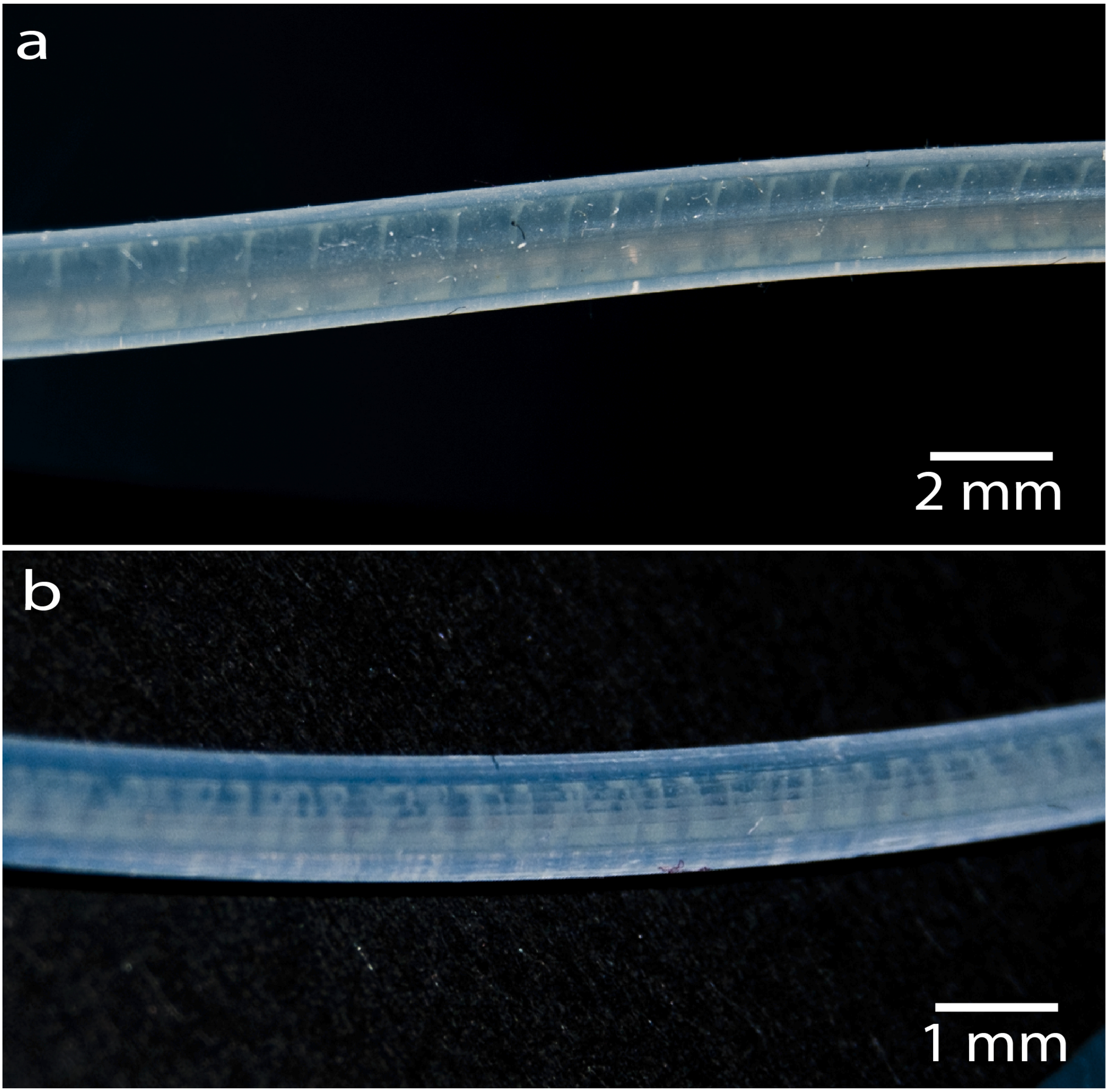

Figure S2. The helical pattern adapts its geometry to the tube radius where it develops. Helical pitches in tubes of 2 mm inner diameter (**a**) are usually larger than those in 1 mm tubes (**b**). Brightness and contrast were adjusted to enhance the image.

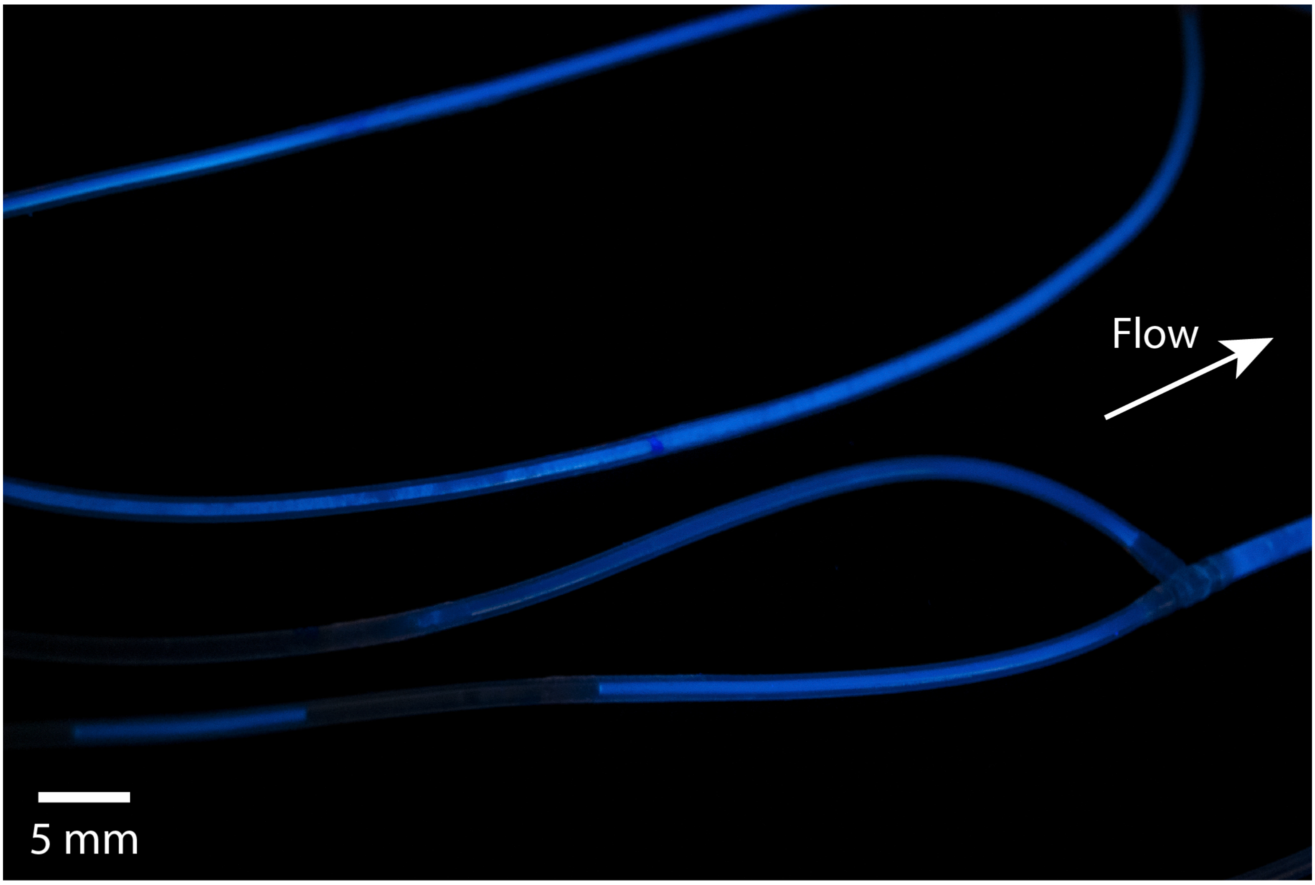

Figure S3. The biofilm expands to fill the length of the tubes they colonize. Colonized zones glow in blue as the result of an autofluorescence phenomenon typical of *Pseudomonas putida (*see reference [1] provided at the end of supplementary material) when exposed to light at 312 nm. Dark zones in the tubes contain air bubbles. Brightness and contrast were adjusted to enhance the image.

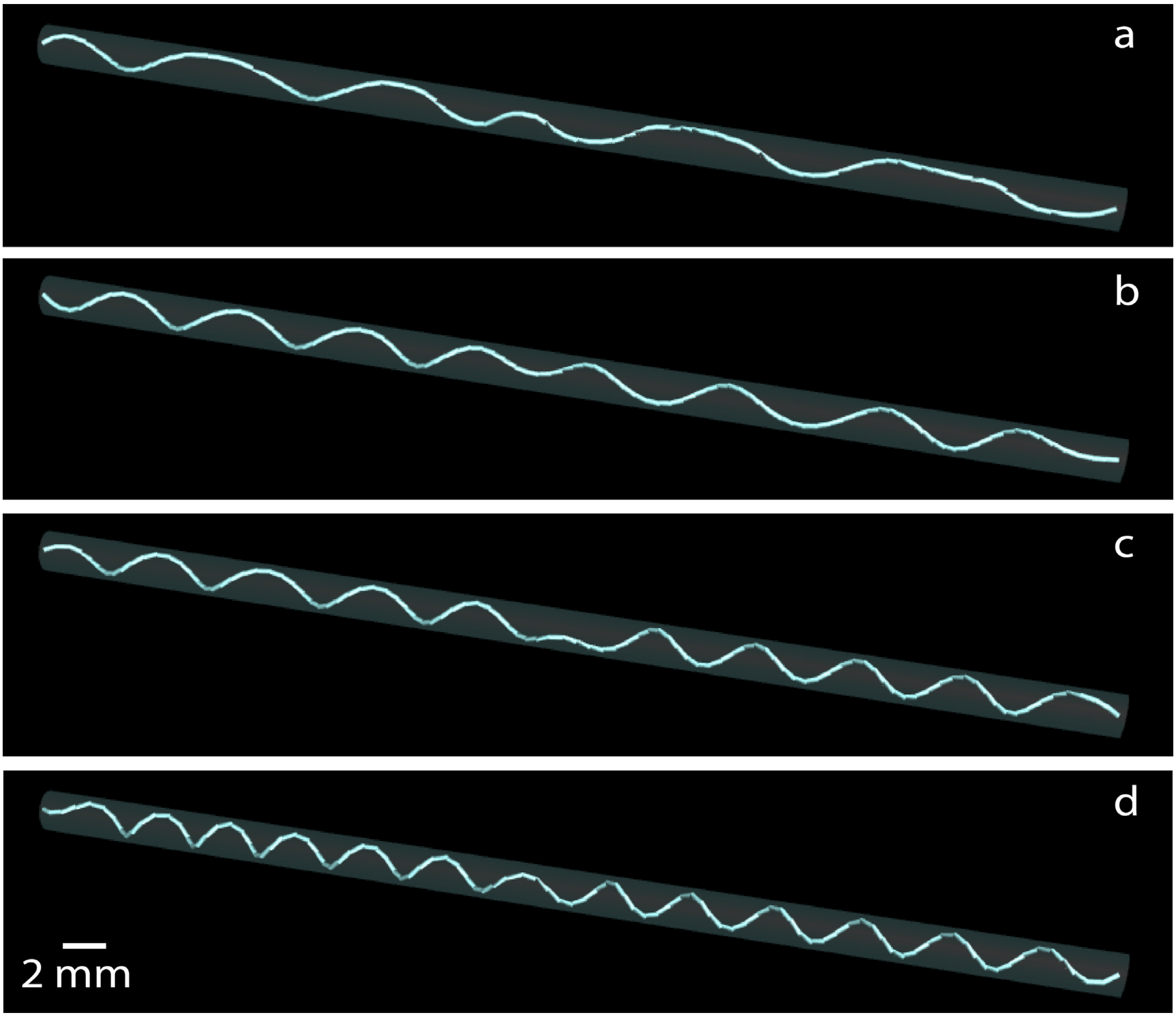

Figure S4. Numerical simulation of helix dynamics using our discrete filament model. As more biomass accrues (a-d), the pitch of a helix diminishes. The length of a complete turn of helix of pitch h and circumference $2\pi r$ is $(h^2 + 4 \pi^2 r^2)^{1/2}$. A thread of length L_h wraps around a tube of radius r and length L_t forming k loops of pitch L_t/k if $L_h^2 \sim L_t^2 + 4 \pi^2 r^2 k^2$. As L_t grows, the helix pitch decreases according to this rule. In our dynamical simulation of filament growth, an imposed twist facilitates a stable evolution avoiding knots and messy structures. The helix pitch for the last image is 5mm.

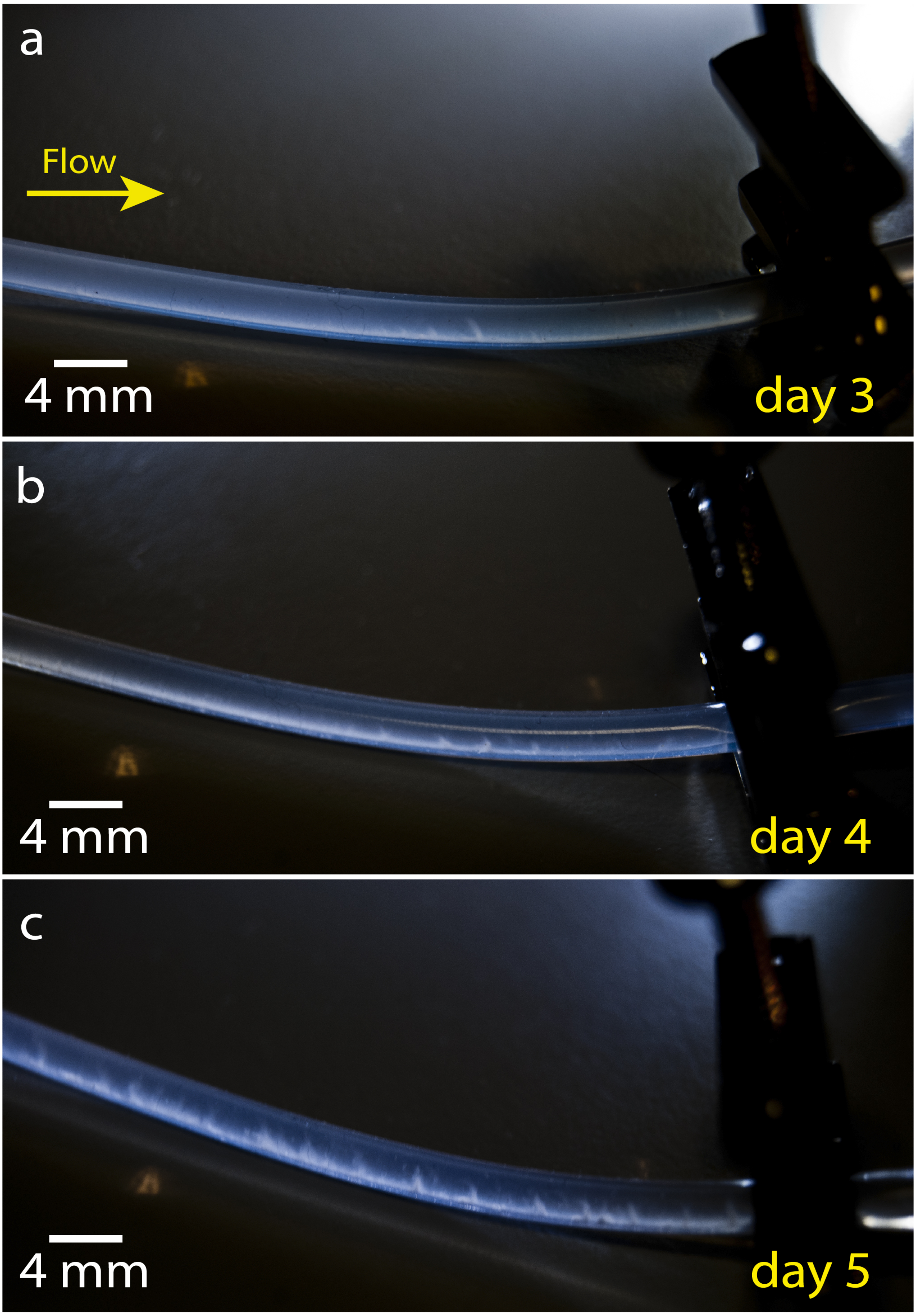

Figure S5. Evolution of a *P. putida* mt-2 biofilm thread formed following a stenosis created by a clamp constraining vertically a 2 mm inner diameter silicone tube at a 0.15 ml/min flow rate as illustrated in Fig 1d in the manuscript. Brightness and contrast were adjusted to enhance the image.

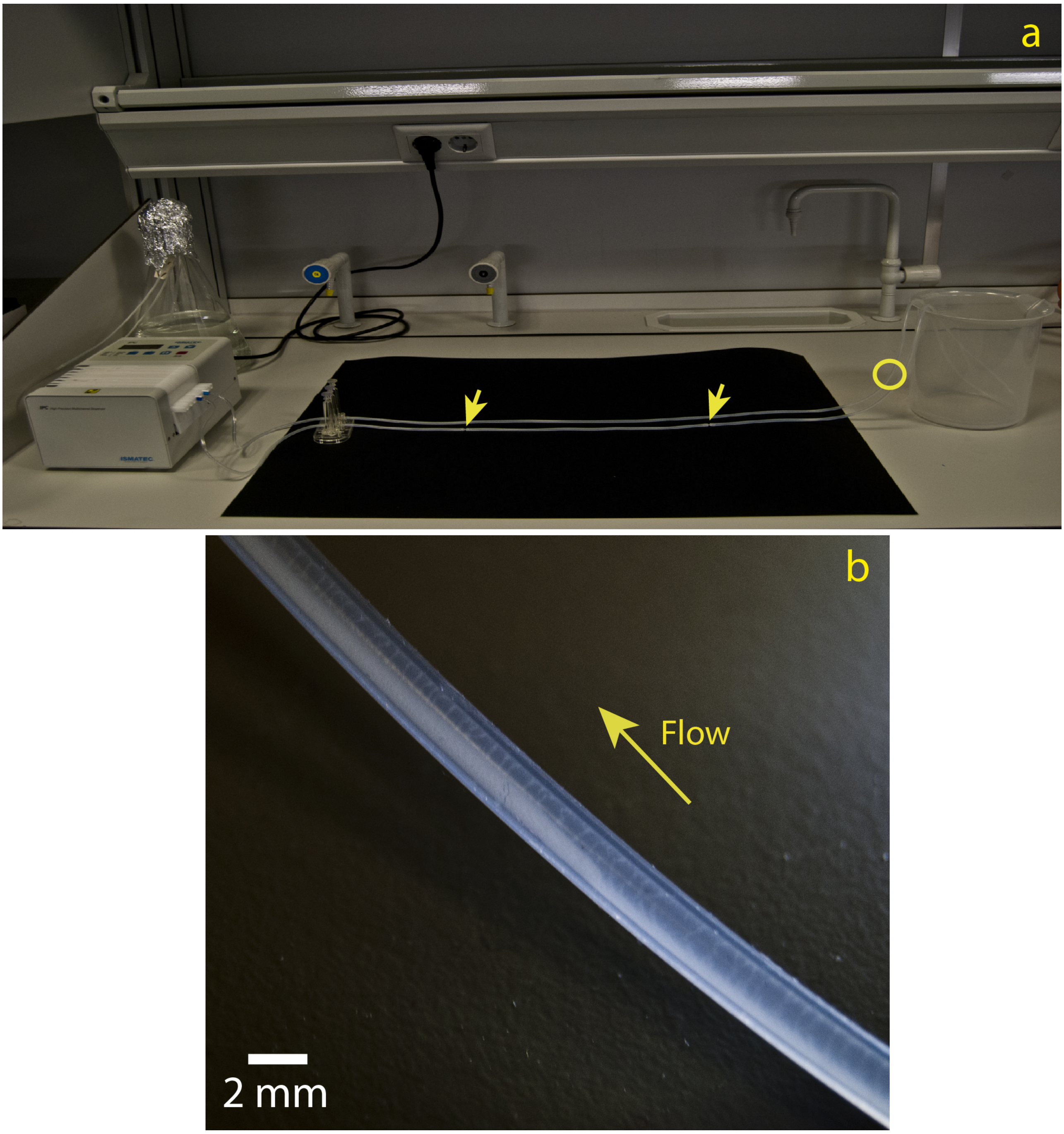

Figure S6. Although stenoses foster nucleation, the coiling effect created at the end of the circuit might perturb the biomass thread by generating twist at its extreme even in absence of them. (**a**) Experimental setup formed by two lines, the first one is perturbed by two adaptors (indicated by yellow arrows) and the second does not have any constriction. (**b**) Tube showing a helix formed by this coiling effect into an unperturbed tube. The inoculated strain was *P. putida* mt-2 and the imposed flow rate was 0.15 ml/min. The photograph was taken at the position of the assembly depicted by the yellow circle in (a). Brightness and contrast were adjusted to enhance the image.

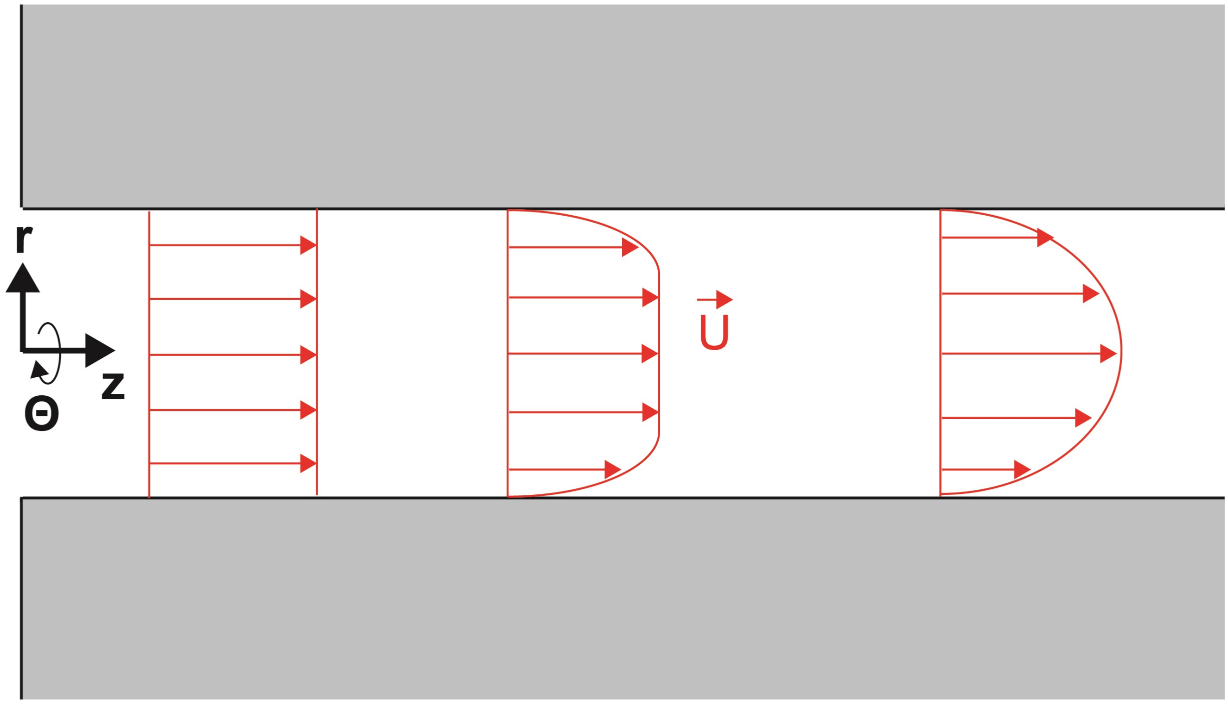

Figure S7. Poiseuille flow profile evolution. The flow evolves from the entrance of the channel to adopt the final laminar steady state profile. The laminar steady flow of an incompressible viscous fluid driven by a pressure drop Δp established between two ends of a straight tube of uniform circular cross-section is described by the final parabolic Poiseuille profile. Writing down the Navier-Stokes equations in cylindrical coordinates (r,Θ,z), the velocity of the fluid **U**=(u_r,u_Θ,u_z) is given by u_r=u_Θ=0 and u_z= Δp ($R^2 - r^2$)/4μ, where R is the tube radius and μ the fluid viscosity. Additional information can be found in reference [2] provided at the end of supplementary material.

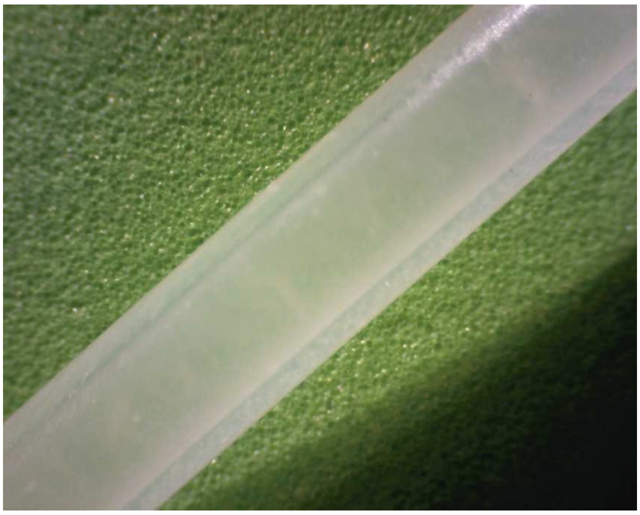

Movie S1 (Download MovieS1_lowq.avi from supplementary material). Accelerated movie (1 second in the movie corresponds to 1.75 hours of experimental time) showing that for a fixed position in the circuit, the biomass thread changes its geometry slowly with time, in a similar way to numerically simulated threads. Note that flow velocities near the walls are much slower than fluid average velocities, already low (about 0.8 mm/s). For high resolution video, visit: http://www.mat.ucm.es/~acarpio/helix/MovieS1.avi

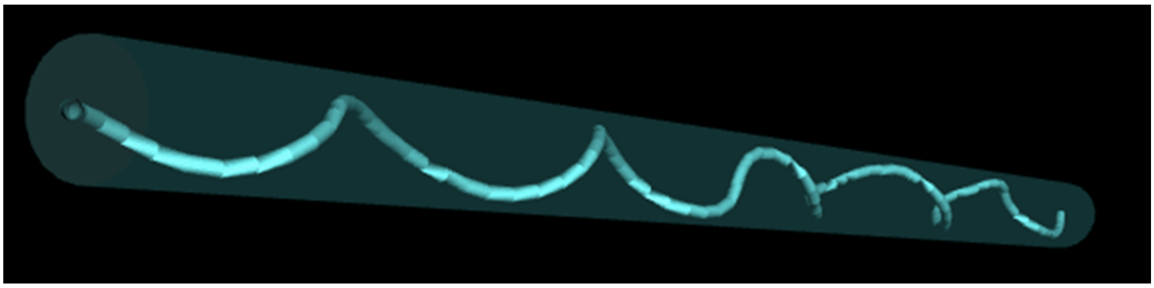

Movie S2 (Download MovieS2.avi from supplementary material). Computational simulation of our discrete filament model showing the emergence of the helical instability, followed by helix coarsening. Still images of this movie are shown in Fig. 6.

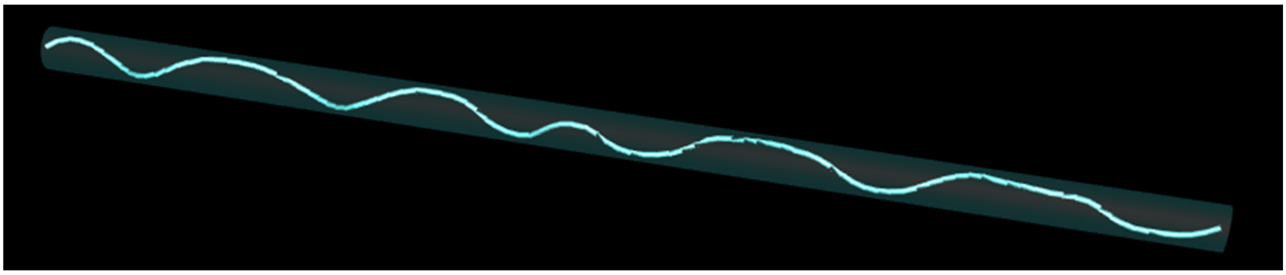

Movie S3 (Download MovieS3.avi from supplementary material). Computational simulation of our discrete filament model showing the reduction of the helix pitch as biomass accrues. Several still images of this movie are shown in Supplementary Fig. S4.

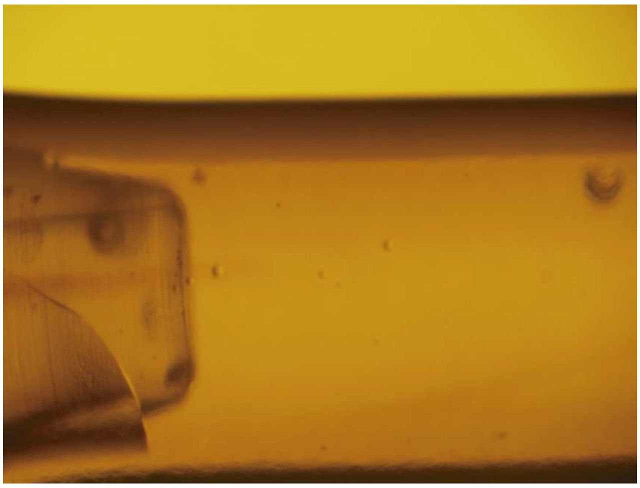

Movie S4 (Download MovieS4.avi from supplementary material**).** Ink assay in a circular adaptor. Streamlines did not show any helicoidal trajectory.

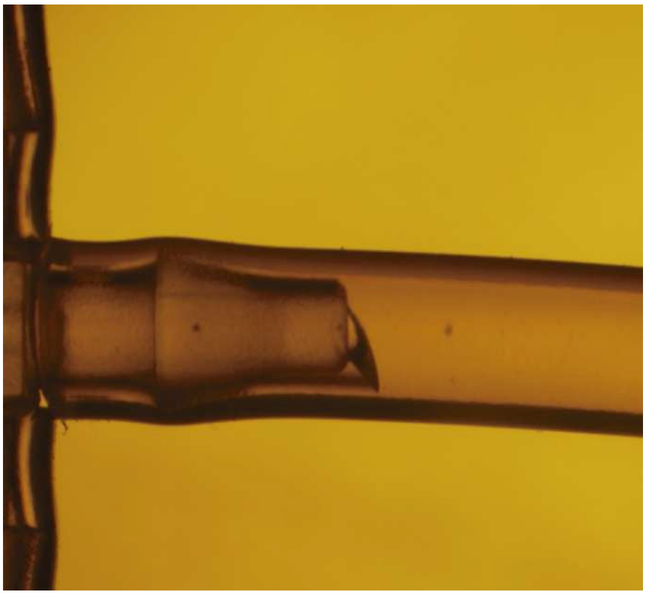

Movie S5 (Download MovieS5.avi from supplementary material). Ink assay in a T-joint adaptor. Streamlines did not showed any helicoidal trajectory.

References

1. Matthijs, S. et al. Siderophore-mediated iron acquisition in the entomopathogenic bacterium Pseudomonas entomophila L48 and its close relative Pseudomonas putida KT2440. Biometals 22, 951–964 (2009).
2. White, F. M. , Fluid Mechanics, McGraw Hill 7th Ed. (2007)
